# A potent neutralizing antibody with therapeutic potential against all four serotypes of dengue virus

**DOI:** 10.1038/s41541-016-0003-3

**Published:** 2017-01-23

**Authors:** Meihui Xu, Roland Zuest, Sumathy Velumani, Farhana Tukijan, Ying Xiu Toh, Ramapraba Appanna, Ern Yu Tan, Daniela Cerny, Paul MacAry, Cheng-I Wang, Katja Fink

**Affiliations:** 1 Singapore Immunology Network, Agency for Science, Technology and Research, Singapore, Singapore; 2 Department of General Surgery, Tan Tock Seng Hospital, Singapore, Singapore; 3 School of Biological Sciences, Nanyang Technological University, Singapore, Singapore; 4 Yong Loo Lin School of Medicine, National University of Singapore, Singapore, Singapore

## Abstract

A therapy for dengue is still elusive. We describe the neutralizing and protective capacity of a dengue serotype-cross-reactive antibody isolated from the plasmablasts of a patient. Antibody SIgN-3C neutralized all four dengue virus serotypes at nano to picomolar concentrations and significantly decreased viremia of all serotypes in adult mice when given 2 days after infection. Moreover, mice were protected from pathology and death from a lethal dengue virus-2 infection. To avoid potential Fc-mediated uptake of immune complexes and ensuing enhanced infection, we introduced a LALA mutation in the Fc part. SIgN-3C-LALA was as efficient as the non-modified antibody in neutralizing dengue virus and in protecting mice while antibody-dependent enhancement was completely abrogated. The epitope of the antibody includes conserved amino acids in all three domains of the glycoprotein, which can explain its cross-reactivity. SIgN-3C-LALA neutralizes dengue virus both pre and post-attachment to host cells. These attributes likely contribute to the remarkable protective capacity of SIgN-3C.

## Dengue: A single vaccine candidate for all strains

An antibody-vaccine candidate has been discovered that neutralizes and confers protection against all four strains of dengue virus. Katja Fink, of the Singapore Immunology Network and Nanyang Technological University, Singapore, describes this as the first time a highly neutralizing antibody has shown efficacy against all four strains. Fink’s team, consisting of scientists from institutions across the country, tested the candidate (SIgN-3C-LALA) in both pre-exposure and post-exposure mouse models. The findings showed it reduced blood-virus levels, protected from lethal infection, and also offered improved safety—without compromising efficacy—the latter by way of intentional mutations to the antibody’s structure. As the global cost of dengue treatment is as high as 9 billion US$ per year, further study is needed to evaluate the suitability of the antibody candidate for the treatment of dengue in humans.

## Introduction

Around 400 million people globally are infected with dengue virus every year, of which about 100 million develop symptoms of varying severity.^1^ The fatality rate of dengue is very low in countries with developed healthcare systems, where patients can be observed and where intravenous fluid replacement regimens can be implemented. However, the economical burden of dengue is high and the global cost of dengue treatment alone has recently been estimated to be US$ 8–9 billion per year.^2^ This number does not include the cost of vector-control measures and time lost at work. A vaccine has recently become available^3^ and has been licensed in a number of countries. However, no significant efficacy has been demonstrated in dengue-seronegative individuals, limiting the implementation largely to endemic countries and the adult population. A specific treatment for dengue has remained elusive so far despite more than a decade of effort to develop a small molecule drug.^4^

Antibodies (Abs) are a potential alternative to small molecules for the treatment of dengue. Many mouse or human monoclonal Abs have been characterized over the past few years, increasing the understanding how Abs neutralize dengue virus. In general, Abs with the most potent in vitro neutralization capacity are serotype-specific.^5–8^ Biologically active Abs target the surface glycoprotein of dengue virus, called E protein. The virus coat consists of 180 copies of the E protein densely packed into 90 E protein dimers.^9^ A number of potent neutralizing Abs target domain III of the E protein that is prominently exposed on the surface of mature virus particles and is therefore easily accessible for Abs.^6,7,10,11^ However, it has emerged more recently that potent human Abs bind to epitopes that include not only the EDIII but also span across EDI and/or EDII. If the epitope includes two or three adjacent E protein dimers, the complex or quaternary epitopes are only present on virus particles. Alternatively, the epitope may be present in recombinantly produced and spontaneously dimerizing E protein, for example if the epitope lies at the interface of an E dimer.^12^ A number of highly neutralizing serotype-specific quaternary, epitope-binding Abs have been described.^5,13,14^ However, not all of these Abs show protective efficacy when tested in mice.^15^

Given the high neutralizing capacity of serotype-specific Abs it is a viable strategy to develop a mixture of Abs, one against each of the dengue serotypes, for therapy. This approach, however, might be costly. In addition, the amount of antibody that needs to be injected with a tetravalent formulation might not be feasible. Abs that potently neutralize all four dengue serotypes could potentially solve these problems, and such cross-neutralizing Abs have been described recently.^12,16^ However, the protective capacity of these Abs is limited^16^ or has not been shown,^12^ respectively.

Besides the cost and potentially limited feasibility of a tetravalent formulation of a dengue therapeutic antibody treatment, the possibility of antibody-dependent enhancement is a major concern. Antibody-dependent enhancement describes the mechanism by which virus complexed with Abs at sub-neutralizing concentration enters the cell via Fc gamma-receptor (FcγR)-mediated endocytosis, resulting in more efficient infection compared to endocytosis of virus alone.^17,18^ This route of immune-complex-mediated infection has been widely documented in vitro and is clinically most relevant in babies born to dengue-immune mothers, whose IgG Abs cross the placenta. While dengue virus (DENV)-specific Abs acquired from the mother are protective for a few weeks after birth, Abs have a limited half-life and the protective capacity is lost once the concentration of the Abs falls below the neutralizing threshold, potentially enhancing a dengue infection that occurs at this time point.^19–21^ In the context of a natural re-infection, where not only Abs but also specific immune B and T cells pre-exist, the implication of antibody-dependent enhancement (ADE) is less clear. Based on the studies in a mouse model, it has been proposed that ADE can be prevented in the presence of a protective T cell response.^22^

We describe here an antibody with potent in vivo efficacy against all four serotypes, both prophylactically and therapeutically. We also provide evidence that an FcγR-binding deficient mutation of the antibody abrogates ADE without compromising its efficacy, addressing the potential safety concerns of a dengue therapeutic antibody.

## Results

### Isolation of a human antibody that binds to intact virus particles of all four dengue virus serotypes and shows high cross-neutralizing activity

**Fig. 1:**
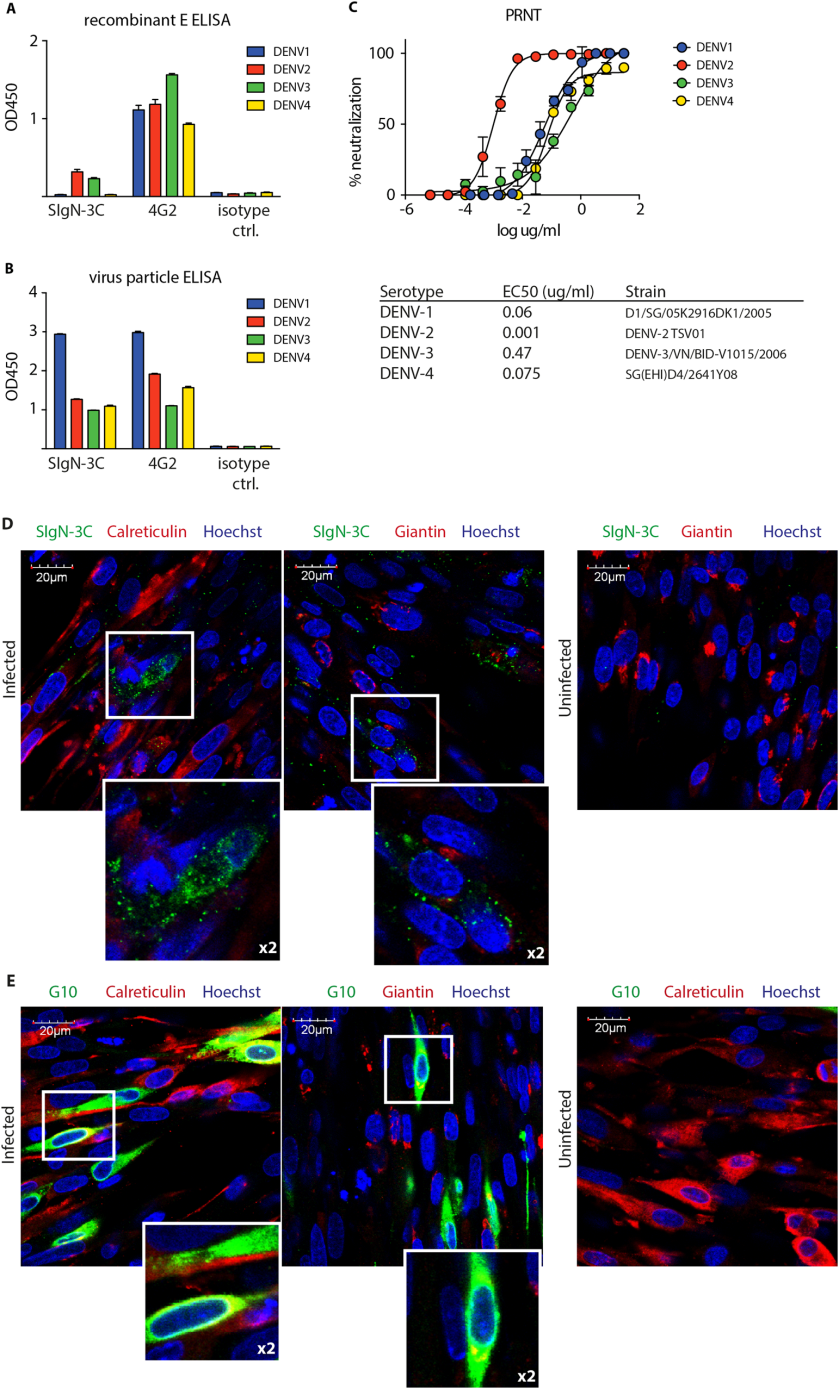
Distinct binding pattern to a conformational epitope and high neutralizing capacity of antibody SIgN-3C. **a** rE ELISA for all four DENV serotype, comparing SIgN-3C to fusion-loop specific Ab 4G2. An influenza-specific antibody was used as an isotype control. **b** Virus particle ELISA for all four DENV serotypes. 4G2 was used as a coating antibody to immobilize non-inactivated virus particles. A and B: bars are means ± SD of triplicates. **c** BHK-21 cell-based PRNT of SIgN-3C for all four DENV serotypes. Each value is the mean ± SD of triplicates and data are representative of at least two individual experiments. PRNT50 values are indicated below the graph. **d**, **e** Immunofluorescence of DENV-2 infected BHK-21 cells. Co-stains with Abs against calreticulin or giantin to assess the binding of SIgN-3C (**d**) or fusion loop-specific antibody G10 (**e**) to E protein in the ER or the trans-golgi network, respectively. Boxed sections are 2x magnified to better show co-localization

Previously, we reported the isolation of a panel of DENV reactive human Abs obtained by single-cell Polymerized chain reaction (PCR) cloning from the sorted plasmablasts of naturally infected dengue patients. Most of these Abs were cross-reactive to all four DENV serotypes and possessed weak to moderate neutralization capacities.^23,24^ We also showed that most Abs bound to recombinant envelope protein (rE). Further studies identified one antibody, SIgN-3C, that exhibited poor rE reactivity but showed significant binding to non-inactivated virus particles (Fig. 1a, b). Of note, virus preparations of individual serotypes can contain different amounts of mature versus immature and different amounts of intact virus particles and binding can therefore not be directly compared between serotypes.

To date, only few dengue cross-reactive virus binding human Abs that are also potent neutralizers across all four serotypes have been described.^25^ When we tested SIgN-3C for its in vitro neutralizing capacity by plaque reduction neutralization assay (PRNT), the antibody demonstrated a high neutralization activity across all four DENV serotypes. The PRNT_50_ values ranged from 0.001 µg/ml (6.8 pM) for DENV-2, 0.06 µg/ml (0.41 nM) for DENV-1, 0.075 µg/ml (0.5 nM) for DENV-4 and 0.45 µg/ml (3.08 nM) for DENV-3 (Fig. 1c).

Immunofluorescence confirmed the selective, conformation-dependent binding pattern that was observed in the Enzyme-linked immunosorbent assays (ELISAs). Antibody SIgN-3C showed a weak and punctate staining of DENV-2 infected BHK21 cells. The fluorescent signal for the antibody did not co-localize with endoplasmatic reticulum (ER) marker calreticulin and golgi marker giantin (Fig. 1d). In contrast, the fusion loop-specific human antibody G10 stained infected cells intensely in both the ER and the golgi network (Fig. 1e).

In summary, SIgN-3C showed binding to intact virus particles, pointing towards a quaternary epitope that is not well preserved in rE preparations, and demonstrated rare serotype-cross-neutralizing capacity.

### SIgN-3C protects mice from lethal DENV-2 infection

**Fig. 2:**
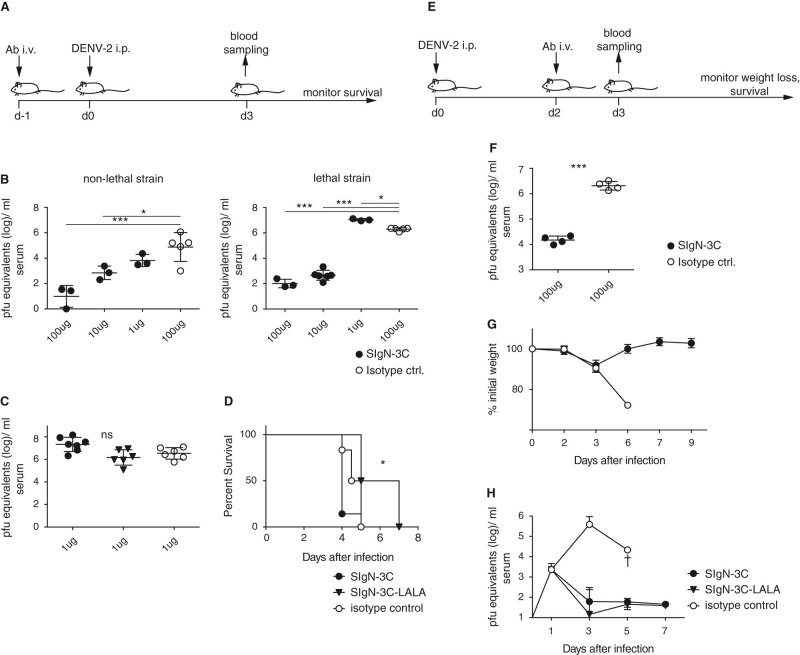
SIgN-3C in vivo efficacy against DENV-2 infection. **a** Scheme for the prophylactic treatment of mice. **b** Viremia in prophylactically treated AG129 mice at day 3 after challenge with non-lethal DENV-2 strain TSV01 and with lethal DENV-2 strain D2Y98P. One way anova *:*p* < 0.05, ***:*p* < 0.001. **c** Viremia at day 3 after challenge in mice treated prophylactically with 1 μg of SIgN-3C, SIgN-3C-LALA or an isotype control. **d** Survival of the mice shown in **c**; Log rank test *:*p* = 0.002 for the comparison between SIgN-3C and SIgN-3C-LALA. An adjusted p value of <0.02 was considered significant. For **c** and **d** data are pooled from two independent experiments with three mice per group each. **e** Scheme for the therapeutic treatment of AG129 mice. **f** Viremia at day 3 after challenge with DENV-2 D2Y98P. student’s t test ***: *p* < 0.0001. **g** Weight loss in IFNAR mice treated therapeutically with SIgN-3C or isotype control. Mice in the isotype control group all had to be euthanized by day 6. **h** Viremia measured at day 1, 3, 5 and 7 after infection with DENV-2 D2Y98P in AG129 mice treated therapeutically with 100 μg SIgN-3C, SIgN-3C-LALA or an isotype control. Mice in the isotype control group all had to be euthanized by day 5. Symbols represent means ± SD, *n* = 4 per group. **b**, **c** and **f**. Each symbol represents one mouse and means ± SD are shown. One way anova test was performed for experiments with more than two groups or a student’s t test for experiments with two groups of mice

**Fig. 3:**
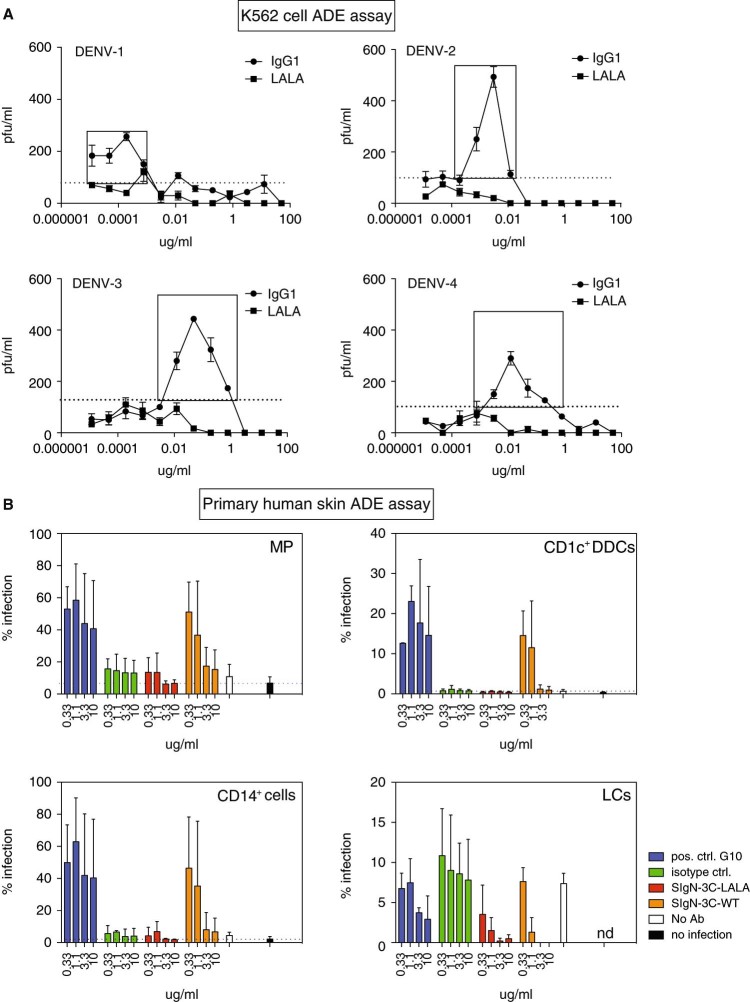
SIgN-3C-LALA shows no enhanced infection in human cells. **a** Antibody-dependent enhancement on K562 cells for SIgN-3C IgG1 (*circles*) and SIgN-3C-LALA (*squares*). Means ± SD are shown. The *dotted line* indicates the limit of detection. Virus in the supernatant of infected K562 cells was quantified in a plaque assay. Enhancement observed with the IgG1 variant (*boxes*) was abrogated with the LALA variant. **b** ADE and neutralization in ex vivo human skin single cell suspensions. CD1c^+^ DDCs: CD1c^+^ dermal dendritic cells; CD14^+^ cells: macrophage precursor/dendritic cells, MP: macrophages, LC: Langerhans cells. *Bars* represent means ± SD of individual values pooled from three independent experiments using skin from different donors (with the exception of G10 and HA, for which the two highest Ab concentrations were only tested in two experiments). The *dashed lines* indicate the background observed in non-infected cells. Different *y*-axis scales for individual cell types were used due to different levels of infection. nd: not detected

Having found a high neutralizing potential of SIgN-3C, we next tested the in vivo efficacy of the antibody. First, SIgN-3C was administered 24 h before infection following a prophylactic protocol (Fig. 2a). The protective capacity of varying doses of SIgN-3C was tested by challenging mice with a non-lethal strain of DENV-2 that was also used for the in vitro assays (TSV01), or with a lethal strain of DENV-2 (D2Y89P). For these experiments, we used mice that lack both the IFN alpha/beta- and gamma-receptor (AG129 mice), a feature that makes them susceptible to DENV infection.^26^ Protection against non-lethal DENV-2 was dose-dependent, and 10 ug Ab per mouse was sufficient to decrease viremia 100-fold (Fig. 3b). Protection against the lethal strain was dose-dependent when 10 or 100 ug Ab were administered, decreasing viremia more than 10,000-fold. However, a slight increase of viremia was observed when only 1 ug Ab was administered (Fig. 2b). Since safety is a key concern for a therapeutic antibody, we introduced two previously described Leu to Ala mutations in the Fc part that abrogate binding to Fc gamma receptors.^27^ The resulting Fc mutant version of 3C (SIgN-3C-LALA) did not increase viremia in mice (Fig. 2c) and seemed to promote survival compared to SIgN-3C when only 1 ug of Ab was administered (Fig. 2d).

The crucial test for an antibody is its efficacy in a therapeutic setting (Fig. 2e). We therefore administered SIgN-3C-LALA 2 days after infection, a time point when the virus is actively replicating and has already reached concentrations of 10^3^–10^4^ pfu/ml, representing a potential treatment scenario of a febrile dengue patient with high viremia.^28^ SIgN-3C-LALA, administered at 100 ug per mouse, was able to efficiently reduce peak virus load in the blood (Fig. 2f), protect mice from weight loss (Fig. 2g) and death (Fig. 2h). Importantly, SIgN-3C-LALA was as efficient as the non-Fc-mutated version of the antibody in protecting mice.

### Abrogation of antibody-dependent enhancement is confirmed in non-differentiated primary human cells

We next assessed whether enhanced DENV infection in human cells was also abrogated by the LALA mutation. We first tested the Abs in the commonly used K562 cell enhancement assay, in which infection is facilitated by FcgRII-mediated uptake of virus-antibody complexes (Fig. 3a). Enhanced infection at low Ab concentrations was seen for SIgN-3C but was completely absent for SIgN-3C-LALA. The infection target cells in humans are dendritic cells and macrophages (MP). Since these cells show differential expression of one or more FcγRs, we next tested enhancement in primary skin cells. We have shown previously that skin dendritic cells and MP are infected efficiently by DENV-2 D2Y98P, and we used this system here to test potential enhancement (Fig. 3b). Fusion loop-specific antibody G10 enhanced infection at all concentrations tested (10–0.33 ug/ml) and was therefore a useful positive control for ADE. Compared to G10, SIgN-3C was enhancing only at lower concentrations. This enhancement, however, could successfully be prevented in dermal dendritic cells (DDCs) (CD1c^+^ DDC), macrophage precursor/dendritic cells (CD14^+^ cells) and MP by using the LALA variant. An influenza-specific antibody was used as isotype control. Interestingly, no enhancement was observed in Langerhans cells, a phenomenon we cannot fully explain yet.

Overall, we show that by using the LALA version of SIgN-3C, ADE could be prevented in the K562 cell line and in freshly isolated, non-differentiated primary cells that are representative, at least in part, of the diversity of DENV target cells in humans.

### Prophylactic and therapeutic treatment with SIgN-3C reduces viremia of all four DENV serotypes in vivo

**Fig. 4:**
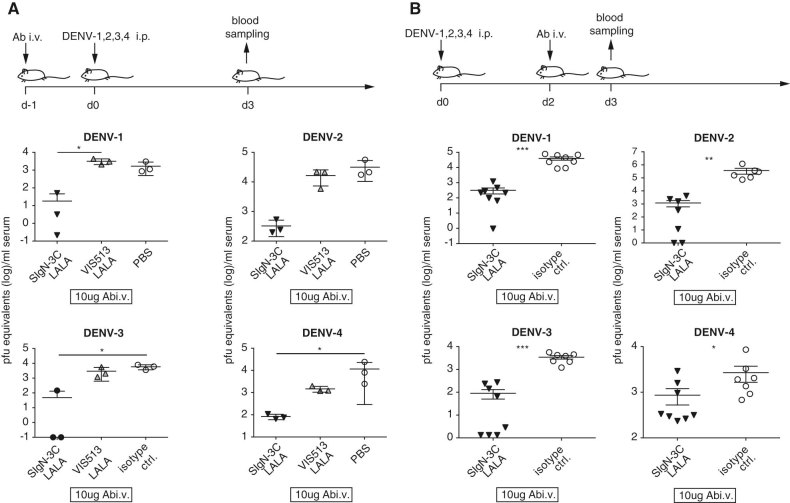
Protection against all four DENV serotypes. **a** Efficacy of SIgN-3C-LALA and 513-LALA in the prophylactic setting. 10 μg of antibody for DENV-1- and DENV-2 and 100 μg antibody for DENV-3 and DENV-4 were given i.v., followed by a challenge with the individual DENV serotypes. Each symbol represents one mouse and means ± SD are shown. Kruskal Wallis test with Dunn multiple comparisons test DENV-4 * = 0.022, DENV-3 * = 0.032, DENV-1 * = 0.033; means ± SD are shown. For testing of DENV-1 and -2 IFNAR mice were used. **b** Efficacy of SIgN-3C-LALA in the therapeutic setting. Results are pooled from two independent experiments with four mice each per group, means ± SEM are shown. Mann–Whitney test; DENV-1: ***:0.0002, DENV-2 ***:0.0012, DENV-3: ***:0.0003, DENV-4: *:0.04

SIgN-3C showed high neutralizing capacity against all four serotypes. However, the PRNT50 values for DENV-3 and DENV-4 were higher compared to those for DENV-1 and DENV-2 (Fig. 1c). It was therefore important to test the protective efficacy of SIgN-3C against all four serotypes. Prophylactic treatment with 10 ug Ab per mouse (for DENV-1 and DENV-2) and 100 ug Ab per mouse (for DENV-3 and DENV-4) reduced viremia significantly for all serotypes (Fig. 4a). A higher dose was chosen for DENV-3 and DENV-4 based on the weaker neutralization capacity of the antibody against these two serotypes. For comparison, we included a previously reported potential therapeutic antibody, VIS513, that was rationally engineered to neutralize all four DENV serotypes.^16^ The results showed that SIgN-3C-LALA was superior to VIS513-LALA in reducing viremia of all serotypes (Fig. 4a).

Therapeutic treatment with SIgN-3C-LALA after infection with DENV-1, 2, 3 or 4 at 100 ug per mouse resulted in a significantly lower peak viremia (Fig. 4b). 100 ug was used for all mice since a higher systemic viral load might need to be neutralized, when the antibody is administered therapeutically. For testing of DENV-1 and 2 IFNAR mice were used due to a temporary unavailability of AG129 mice. IFNAR mice develop slightly lower viremia compared to AG129 mice but show the same virus kinetics, with viremia peaking at day 3 after infection. DENV-1, 3 and 4 strains used in this study are not fatal in AG129 and IFNAR mice and hence survival could not be addressed for these serotypes.

In summary, SIgN-3C showed protective capacity against all four DENV serotypes at a concentration of ≤5 mg/kg, which is unmatched by any other reported serotype cross-reactive antibody.^16,29,30^

### 3C neutralizes DENV pre and post-cell attachment

**Fig. 5:**
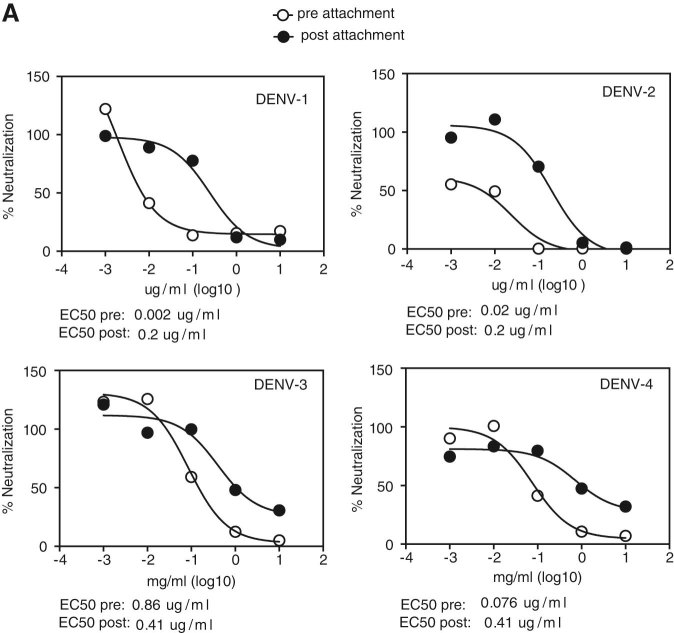
Mode of action of antibody SIgN-3C-LALA. **a** Pre and post-attachment neutralization assay with U937-DC-SIGN cells. EC50 values for the pre and post-setup are shown below the graphs. Symbols are means of duplicates and results are representative of one experiment out of two

Abs can neutralize viruses by various mechanisms. For direct inhibition of virus attachment to host cells, antibody binds to and blocks the host cell receptor-binding site on the virus. If the antibody is able to compete with virus-host cell receptor binding, infection can also be blocked post virus attachment to cells. To test the capacity of SIgN-3C-LALA to block pre and post-cell attachment of DENV, we either incubated virus and antibody before adding the virus -antibody complexes to U937-DC-SIGN cells, or allowed virus to bind to the cells first before adding the antibody (Fig. 5a). Of note, this assay compares between binding of SIgN-3C to virus pre-attachment and post-attachment to host cells but it does not allow to make conclusions on the ability of antibody to block fusion in the endosome.

We found that pre-attachment neutralization was, expectedly, more efficient. However, SIgN-3C-LALA was also able to neutralize all four DENV serotypes post-attachment.

### The binding site of SIgN-3C includes conserved amino acids in two domains of the E protein

**Fig. 6:**
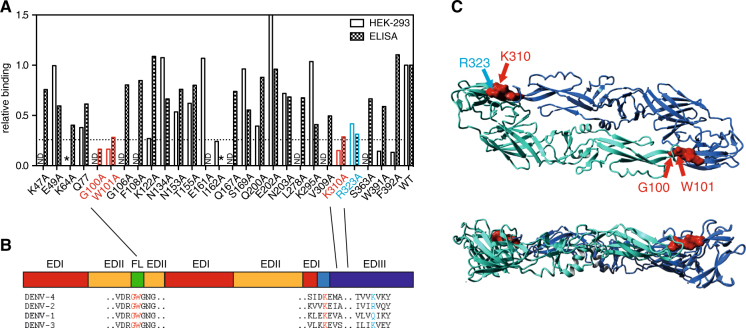
Binding sites of SIgN-3C. **a** Epitope mapping using mutated E proteins in ELISA (*filled bars*) and mutated E protein expressed on the surface in HEK-293T cells (*empty bars*). Bars are means of duplicates and results are representative of three and two independent experiments, respectively. *Red bars* indicate likely binding sites of SIgN-3C as shown by a 75% reduction of binding in one and/or both assays. *Blue bars* indicate a possible binding site shown by 70% reduction in binding. ND: not done. *: misfolded protein in either the HEK or ELISA assay, I162 was therefore not included as part of the epitope. 75% reduction is indicated by the *dotted line*. **b** Illustration of the proposed binding sites on the soluble part of the E protein. The individual domains of the E protein are indicated in different colors according to.^49^ *Red*: EDI, *yellow*: EDII, *blue*: EDIII, *light blue*: EDI-EDIII linker, *green*: FL (fusion loop). Sequence alignment of the binding sites for all four DENV serotypes is shown with the binding sites highlighted in *red* and *blue*. **c** Illustration of the proposed binding sites on the dimeric structure of the E protein in top-down and side view. The individual residues are indicated with *arrows* in the turquoise E monomer

To obtain a basic and non-exhaustive map of the binding site of SIgN-3C, we employed a small library of alanine replacement mutants of the soluble part of the DENV-2 E protein. The amino acids were chosen based on their exposure to the surface of the E dimer.^31^ Since SIgN-3C does not bind to E protein monomers, we used a sandwich ELISA approach that immobilizes V5-tagged E protein on ELISA plates via an anti-V5 tag antibody. This approach increases the concentration of E protein and promotes the immobilization of dimers.^32,24^ In addition to the ELISA approach, we expressed a subset of the same alanine substitution mutants as full-length prM-E proteins in HEK293T cells and screened for binding of the antibody on the surface of cells. The screens identified three alanine substitution mutants, G100A, W101A and K310A to which binding of SIgN-3C was reduced consistently to ≤20% (Fig. 6a, indicated in *red*). All three amino acids were completely conserved across the four DENV serotypes, providing an explanation for the cross-reactivity of SIgN-3C (Fig. 6b). An additional non-conserved residue, R323A, abrogated binding in both assays to 30–40% and is therefore a likely binding site (Fig. 6a, b, indicated in *blue*). The I162A mutation abrogated binding in the HEK cell assay but the soluble protein could not be produced consistently for the ELISA, suggesting that it might be mis-folded.

The potential binding sites of SIgN-3C illustrated on a published E dimer structure showed that the four amino acids described in Fig. 6a, b comprised a cluster of G100 and W101 on one monomer and K310 and R323 on the other monomer (Fig. 6c). Antibody SIgN-3C has a remarkably long heavy chain CDR3 (Table 1), potentially allowing the antibody to cover an epitope that spans over more than one dimer. The 12% mutation rate of the heavy chain (12 out of 98 amino acids) indicates a considerable level of affinity maturation and suggests that the antibody could have been affinity-matured between the first and subsequent infection.

**Table 1:** Variable region analysis of antibody SIgN-3C

	V gene	D gene	J gene	V region AA mutations	CDR3	CDR3 length
Heavy chain	IGHV1-46	IGHD3-22	IGHJ5-02	12/98	ARGGRALFYDSYTTPRDGGSWWFDP	25
Light chain	IGKV1-33	–	IGKJ5	8/94	QQFDDLPIT	9

## Discussion

To date, the occurrence of cross-reactive human Abs against dengue virus that are also potent neutralizers has rarely been described. After a second or subsequent infection, the plasmablast response in patients produces almost exclusively serotype-cross-reactive Abs.^23,33,34^ These plasmablasts are mostly re-activated memory B cells that bind to serotype-conserved epitopes. One of the dominant epitopes amongst plasmablasts is the fusion loop, which is essential for virus fusion with the host membrane. The fusion loop is 100% conserved across all DENV serotypes, and even across all flaviviruses. While a majority of Abs in patients bind to the fusion loop,^35,36^ these Abs do not have a high neutralizing capacity and are not protective in mice.^32^ The poor biological activity of serotype-cross-reactive Abs is not surprising given the definition of a virus serotype, which is based on the reactivity and protective capacity of homologous versus heterologous serum.^37^ Accordingly, individuals infected with one DENV serotype are protected against that particular serotype but not against the other three.^38^ However, in apparent contradiction to the serotype definition, rare Abs with high cross-neutralizing capacity do exist within the large pool of cross-reactive Abs produced during acute secondary DENV infection.^12^

The antibody SIgN-3C that we describe here showed uncommon characteristics: it bound to E protein dimers or multimers and to intact virus particles (Fig. 1) and it can block infection both pre and post-attachment of virus to the host cell (Fig. 4). We speculate that SIgN-3C can block virus fusion in endosomes given its potential capacity to lock dimers across the fusion loop and EDIII (Fig. 5c). Importantly, SIgN-3C and the SIgN-3C-LALA variant show protective capacity in vivo against all four DENV serotypes (Fig. 3). This is relevant in the light of discrepancies between in vitro neutralization and in vivo protective capacity. For example, antibody VIS513 shows neutralizing titers that are very similar to SIgN-3C, yet its protective capacity in vivo is low (Fig. 3a). The epitope of VIS513 is located exclusively in domain III, which might more easily lead to the generation viral escape variants compared to an epitope that spans across several domains. Complex viral epitope-specific Abs might also be less likely to bind to host or ‘self’ structures. The epitope described here is incomplete since our screen was not exhaustive. Binding to the fusion loop and K310 has been described for several human Abs and binding to these residues alone cannot explain the remarkable efficacy of SIgN-3C. We undertook several attempts to generate viral escape mutants for the purpose of epitope mapping but could not recover any escaped viruses. Interestingly, viral escape mutants were described under pressure of antibody 1A1D-2, which binds to residues K310 on EDIII.^10^ The absence of escape mutants for SIgN-3C thus suggests that the fusion-loop and potentially other conserved residues that are essential for the integrity of the virus structure are critical components of the neutralizing epitope. Further work to identify the detailed epitope of the antibody is currently ongoing.

Since no clinical trials with dengue therapeutic Abs have been conducted so far it is still uncertain to which extent the findings in mice can be extrapolated to patients. This is particularly so for Fc region-dependent activities. Humans express FcgRIIa, which is the main driver of ADE.^39^ In turn, FcgRIIb seems to be able to inhibit ADE.^40^ Mice only express FcgRIIb yet ADE is readily observed, suggesting that different mechanisms could account for ADE in mice. Despite these differences, human IgG Abs bind to mouse Fc receptors and the LALA mutation abrogates this binding, validating the mouse model for the testing of Fc-modified Abs to at least FcϒRs. To confirm the ability of SIgN-3C–LALA to block ADE in a relevant human system, we used primary skin cells that contain different types of dendritic cells and macrophages that are representative of human cells susceptible to viral infection. These cells also represent the first target cells after the virus is released into the dermis during a mosquito bite.^41^ Interestingly, the level of ADE differs between different dendritic cells and monocytic/macrophage cells, highlighting to the complexity of ADE in humans. Langerhans cells do not show any ADE but instead function as target cells to assess neutralization, confirming the efficacy of SIgN-3C in a primary human cell assay. As expected, the fusion loop-specific Ab G10 is less neutralizing than SIgN-3C and SIgN-3C-LALA (Fig. 3b).

Since the efficacy of SIgN-3C-LALA was similar to the efficacy of wild-type SIgN-3C in mice, antibody-dependent cytotoxicity does not seem to play a major role in the clearance of DENV. The half-life of LALA and wild-type antibody versions of two Abs, anti-OX40L and anti-HIV antibody b12, was compared previously in cynomolgous macaques by two independent groups, and both report that the LALA mutation did not significantly reduce the antibody half-life.^42,43^ Given these findings we propose that a LALA-variant is a viable strategy for the development of a therapeutic antibody for dengue.

In summary, SIgN-3C is a cross-reactive DENV antibody with potent in vitro and in vivo efficacy against all four DENV serotypes. The application of this antibody can be both prophylactic and therapeutic, since a potential ADE risk can be minimized with the modification of the antibody Fc part, without compromising efficacy.

## Materials and methods

### Viruses used in the study

All virus stocks were produced in C6/36 cells (ATCC). This and all other cell lines used in this study were regularly tested to be free of mycoplasma. Infected cell culture supernatants were collected, centrifuged at 2000 g to remove cell debris and virus was aliquoted for storage at −80 °C. The following strains were used in this study: DENV-1-D1/SG/05K2916DK1/2005 [EU081234.1], DENV-1-WestPac74 (U88535.1), DENV-1-08K3126 (unpublished, received from the Environmental Health Institute EHI, Singapore), DENV-2-TSV01 (AY037116.1), DENV-2-DENV-2/SG/D2Y98P-PP1/2009 (JF327392.1), DENV-3-VN32/96 (EU482459), DENV-3 D3/SG/05K4141DK1/2005 (EU081214.1), DENV-4-SG(EHI)D4/2641Y08 (HQ875339.1), DENV-4-TVP360/341750 (GU289913.1).

#### ELISA

For whole virus particle ELISA Maxisorp plates (Nunc) were coated with 4G2 antibody and virions were captured from infected C6/36 cell supernatants. The following virus strains were used: DENV-1-05K2916, DENV-2-TSV01, DENV-3-VN/32 and DENV-4-2641Y08. For rE ELISA, Maxisorp plates (Nunc) were coated with 150 ng of purified rE protein in 100 ul coating buffer at 4 °C overnight. rE protein was produced in S2 cells as described previously.^44^ The sequences were derived from DENV-1-05K2916, DENV-2-TSV01, DENV-3-08K4141 and DENV-4-2641Y08. Abs were added at 1 μg/ml and binding was detected by adding anti-human IgG-HRP. TMB substrate was used for the color reaction.

#### ADE assays

Dengue virus seed stocks of all four serotypes were diluted to achieve an multiplicity of infection (MOI) of 0.1 and added to Abs, which were diluted four-fold over 12 dilutions starting from 30 ug/ml, and incubated at 37 °C for 1 h. K562 (ATCC) cells were added to the virus-antibody suspensions and incubated at 37 °C for 2 h. The cells were then washed with 1× PBS twice and re-suspended in 350 μl of RPMI + 2% FBS. After incubation at 37 °C for 72 h, K562 cells were removed by centrifugation at 8000 g and the supernatant was added to BHK cells in triplicates at 100 μl per well as per PRNT assay. The virus strains used are the same as used in the PRNT assay.

The skin cell preparation and infection protocol have been described previously.^41^ Single cell suspension of normal skin obtained from mastectomy surgery were prepared and infected with DENV-2 D2Y98P at an MOI of 2 for 24 h. Infection in the individual cell types was assessed by flow cytometry using Abs to surface markers to identify dendritic cells and MP,^41^ and an Ab for E protein stained intracellularly (Ab 4G2, hybridoma purchased from ATCC) to identify infected cells. Healthy human skin tissue was obtained from mastectomy surgery. The study was approved by the National Health Group Domain Specific Review Board (NHG DSRB 2015/00725). Patients gave written informed consent.

#### Neutralization assays

BHK-21 cells (ATCC) were seeded at 1 × 10^5^cells/ml in 0.5 ml per well of a 24-well plate and incubated at 37 °C with 5% CO_2_ overnight. Purified Abs were diluted four-fold over 6 dilutions, starting from 30 to 0.03 ug/ml and then added to the virus suspension. Virus-antibody mixtures were incubated at 37 °C for 1 h. Infection was carried out by adding 100 μl to each well in triplicates for each antibody dilution and incubating at 37 °C for 1 h, with rocking every 15 min. An overlay consisting of 0.8% Methylcellulose (Aquacide II, Calbiochem) in RPMI + 5% FCS was added 1h post-infection and the plates were incubated at 37 °C for 4.5 days. The overlay was discarded and monolayer was stained with crystal violet + 0.8% PFA by incubating at RT with rocking overnight. Plates were washed with tap water and plaques were enumerated by eye. PRNT_50_ is defined as the concentration of antibody, which results in a reduction of plaques by 50%, determined by applying a three-parameter non-linear curve fit in GraphPad Prism. The following viruses were used for PRNT: DENV-1-D1/SG/05K2916DK1/2005 [EU081234.1], DENV-2-TSV01 (AY037116.1), DENV-3-VN32/96 (EU482459) and DENV-4-SG(EHI)D4/2641Y08 (HQ875339.1).

Pre and post-attachment neutralization assays were performed as described previously^45^ using U937 cells (ATCC) stably transfected with DC-SIGN. For the pre-attachment assay, antibody was diluted ten-fold from 10 to 0.001 μg/ml, mixed with a constant amount of virus and incubated at 4 °C for 1 h. Antibody-virus mix was added to pre-chilled U937-DC-SIGN cells for 1 h at 4 °C. Cells were then washed three times with medium before incubation for 48 h at 37 °C. For the post-attachment assay, cells were pre-chilled and the same constant amount of virus as for the pre-attachment assay was added to cells. After incubation for 1 h at 4 °C, cells were washed three times with medium and antibody diluted ten-fold from 10 to 0.001 μg/ml was added. Cells were then washed three times with medium before incubation for 48 h at 37 °C. The flow cytometry-based method to detect infected cells with Abs against E and NS1 protein was used as a readout.^46^ The same virus strains as for the PRNT were used, except for DENV-1, for which DENV-1-08K3126 was used in the pre-post-neutralization assay.

#### Epitope mapping

Cell-based assay: Codon optimized DENV-2 (strain FGA/02),^47^ was synthesized by Genescript and cloned into plasmid cDNA3.1-myc. DENV-2 E mutants were produced with a QuikChange Site-Directed Mutagenesis Kit (Agilent). HEK293 cells (ATCC) were seeded in 24well plates, transfected 24 h later with 1 ug plasmid and surface stain was performed with SIgN-3C 2 days post transfection. Anti-myc tag antibody was used to normalize the different expression levels of individual mutants.

The epitope was visualized on a published DENV-2 dimer structure (PDB ID 1OAN) using Yasara software (http://yasara.org).

#### Immunofluorescence

BHK-21 cells were seeded in ibidi slides (15 u-slide angiogenesis, ibidi) and infected with DENV-2 (TSV01) at MOI 1. 48 h post-infection, the cells were fixed with 4% PFA for 20 min at room temperature (RT), permeabilized with 1× PBS + 0.05%Triton X-100 for 15 min at RT and blocked with 1× PBS, 1% BSA for 1h at RT. Cells were then incubated with monoclonal Abs SIgN-3C (2 ug/ml) or rabbit anti-calreticulin (Abcam Ab2907) (1:100) or rabbit anti-giantin (Abcam Ab37266) (1:1000) for 1 h at RT. Secondary Abs goat anti-rabbit AF568 and goat anti-human AF488 (MolecularProbes) were used at 1:2000 dilution. Hoechst 33342 (Molecular Probes) was added for 5 min at RT. All washes were performed with PBS for 10 min at RT. Antibody binding was visualized with an Olympus confocal microscope at 100X magnification, using oil.

#### Mouse experiments

AG129 mice were used unless stated otherwise. AG129 mice were purchased from B&K Universal, UK. IFNAR mice were purchased from The Jackson Laboratories. 6–8 week old male and female mice were used for all experiments. Treatment and control groups were age and sex-matched. Of note, consistent weight loss was only observed in IFNAR but not in AG129 mice and the former were therefore used to quantify weight loss. Antibody was injected intravenously via the retro-orbital route at indicated doses. Virus was injected intraperitoneally. The following virus strains and doses were used: DENV-1 (08K3126, 106 pfu), DENV-2 (D2Y98P, 106 pfu, and TSV01, 106 pfu), DENV-3 (VN32/96, 1.5 × 106), and DENV-4 (TVP360, 3 × 106 pfu). Antibody VIS513-LALA was produced according to published sequences. 3 days post-infection, mice were bled and viremia was determined by real-time PCR as described previously.^48^ All mouse experiments were conducted according to the rules and guidelines of the Agri-Food and Veterinary Authority and the National Advisory Committee for Laboratory Animal Research, Singapore. The experiments were reviewed and approved by the Institutional Review Board of the Biological Resource Center, Singapore (Institutional Animal Care and Use Committee; protocol #151099).
