# Publisher Correction: A potent neutralizing antibody with therapeutic potential against all four serotypes of dengue virus

**DOI:** 10.1038/s41541-018-0044-x

**Published:** 2018-02-01

**Authors:** Meihui Xu, Roland Zuest, Sumathy Velumani, Farhana Tukijan, Ying Xiu Toh, Ramapraba Appanna, Ern Yu Tan, Daniela Cerny, Paul MacAry, Cheng-I Wang, Katja Fink

**Affiliations:** 1 Singapore Immunology Network, Agency for Science, Technology and Research, Singapore, Singapore; 2 Department of General Surgery, Tan Tock Seng Hospital, Singapore, Singapore; 3 School of Biological Sciences, Nanyang Technological University, Singapore, Singapore; 4 Yong Loo Lin School of Medicine, National University of Singapore, Singapore, Singapore

**Correction to**: *npj Vaccines* 10.1038/s41541-016-0003-3, published online 23 January 2017

The original version of the Article contained errors in the display of the labels in Fig. 6b. These errors have now been corrected in the HTML and PDF versions of the paper.

